# Childhood Immunity and Infections: Time to Consider Endothelial Cells and Platelets

**DOI:** 10.3390/children9060841

**Published:** 2022-06-06

**Authors:** Danilo Buonsenso

**Affiliations:** 1 Department of Woman and Child Health and Public Health, Fondazione Policlinico Universitario A. Gemelli IRCCS, 00168 Rome, Italy; danilobuonsenso@gmail.com; Tel./Fax: +39-(0)-630154390; 2 Global Health Research Center, Università Cattolica del Sacro Cuore, 00168 Rome, Italy

The immune system was, and still is, the protagonist of this pandemic. Initially, COVID-19 was defined as a cytokine storm, where the immune system played a fundamental role in the pathological expression of the disease [1]. Subsequently, the difference in clinical severity of COVID-19 between adults and children aroused much interest and still pointed at the immune system as a determinant factor of these differences [2]. We now know that, in children, a higher expression of innate immune responses, a well-defined higher expression of IFN-gamma in the upper airways in response to SARS-CoV-2 infection [3], and an expansion of T-regulatory compartments [4] play a role in the differential severity of disease expression, although other factors may play a role. In particular, an important difference between adults and children is the higher development of thromboembolic events in adults during or after COVID-19 [5]. In the Multisystem Inflammatory Syndrome (MIS-C), an exaggerated immune response leads to the development of a severe disease characterized by high fever, inflammatory markers, and a procoagulation state [6]. Moreover, growing evidence supports a primary role of the immune system and endothelial/coagulation pathways in the genesis of Long COVID in a subgroup of patients [7]. Last, differences in immune responses to vaccinations and infection are creating extremely variable susceptibility to reinfections in people of all age groups [8].

Although in each of these steps the peculiar immune mechanisms have been better characterized, experts are increasingly recognizing a common actor involved in all these moments: the endothelium. While COVID-19 was initially considered a respiratory disease, there is now evidence that this is a systemic inflammatory condition characterized by viral invasion potentially in most parts of the organism and endothelial inflammation. Laboratory parameters of endothelial phlogosis and activation of the coagulation cascade is a hallmark of the disease, which has thromboembolic events and myocarditis as a cornerstone of the infection [6,7,8]. Conversely, these events are much less frequent in children during COVID-19. In the rare circumstance when children develop MIS-C after SARS-CoV-2 infection, D-Dimers are characteristically high, children have myocardial dysfunction or coronary involvement, and are mostly treated with anti-coagulants or anti-aggregants [6]. In Long COVID, chronic inappropriate endothelial and platelet activation, lung hypoperfusion and microclots have been clearly demonstrated [7,9]. Last, a specific group of people (young males) in a minority of cases can develop myocarditis as the main complication of mRNA anti-COVID vaccination [8], suggesting again a close relationship with cells interfacing with the blood.

Taken all together, these observations reinforce recent interest toward the endothelium playing an active and pivotal role in immunity, responses to infections, and homeostasis of the organisms, rather than a simple bystander transporting blood around the body.

## 1. Immunological Roles of the Endothelium

Endothelial cells (ECs) are able to attract immune cells to sites of inflammation or injury [10]. However, ECs also have a role in antigen presentation and are able to modulate immune responses, with data proving immunometabolism and innate immune memory of ECs [11]. Physiologically, ECs express MHC I (Major histocompatibility class I) molecules and PPRs (pattern-recognition receptors) which detect PAMPs (pathogen-associated molecular patterns) and, in case of inflammatory stimuli, can shift to a pro-inflammatory and pro-coagulatory state, express MHC II molecules, upregulate adhesion molecules for immune cells and facilitate transmigration to underlying tissues, and secrete pro-inflammatory cytokines and chemokines [12].

Fascinatingly, ECs are able to perform the so-called endothelial-to-mesenchymal transition (EndMT), which allows ECs to differentiate into other cell types including chondrocytes, osteoblasts, and fibroblasts, contributing substantially to inflammation-induced fibrosis [10]. In addition, ECs also undergo structural changes with capillary and venule remodeling during chronic inflammation, where the newly formed vessels become permanent and lead to a further influx of immune cells [13].

Recent interesting studies also demonstrated that ECs have many innate immune functions and express gene signatures indicative of phagocytosis or scavenging, antigen presentation, and immune cell recruitment [14]. Specifically, they have functions that macrophages express, including cytokine secretion, phagocytic function, antigen presentation, pathogen-associated molecular patterns-, and danger-associated molecular patterns-sensing, proinflammatory, immune-enhancing, anti-inflammatory, immunosuppression, migration, heterogeneity, and plasticity [11]. Moreover, ECs behave as innate immune cells also in metabolic cardiovascular diseases, other sterile inflammations, and cancers. These concepts are extremely relevant in the case of chronic inflammatory states, which have a known role in cancerogenesis [14].

Jacob Amersfoort and colleagues also proposed and an immunomodulatory function of ECs: in the lung and liver they mediate the balance between tolerance and inflammation, in the kidney and liver they closely interact with resident immune cells, in the brain, they form a tight and low immunomodulatory barrier to minimize infiltration of the tissue parenchyma [14].

Last, ECs have active interactions with pathogens, not only through adhesions molecules, but also by induction of mechanisms that can restrict the growth of micro-organisms (some proven ones regard *T. gondii*, *S. aureus*, Rickettsia, and *M. tuberculosis*), but at the same time also represent an important replicative niche for a subset of viral, bacterial, and parasitic organisms that are present in the blood or lymph [15]. More recently, endothelial cell infection and endotheliitis have been documented through histopathological studies during COVID-19 [16].

## 2. Immunological Roles of Platelets

Despite their small size and anucleate status, platelets have diverse roles in vascular biology that extend beyond their better-established role in thrombosis [17]. There is currently agreement that platelets are also immune cells that initiate and accelerate many vascular inflammatory conditions. There are several known platelet-derived inflammatory mediators and immune modulators that make platelets both an anti-inflammatory or proinflammatory element [17].

Interestingly, platelets can also actively interact with pathogens. Viral particles of various blood-borne pathogens such as HIV, dengue, or even respiratory viruses such as influenza have been demonstrated inside human platelets. The presence of internalized viral particles, thrombocytopenia, and thrombosis implicates platelets as active participants in immunity during viral infections [18]. Recent studies have revealed that platelets exhibit several pattern recognition receptors (PRR) including those from the toll-like receptor, NOD-like receptor, and C-type lectin receptor family and are first-line sentinels in detecting and responding to pathogens in the vasculature. The role of platelet-derived mediators and platelet interaction with vascular and immune cells in protective and pathophysiologic responses has been characterized in cases of dengue, influenza, human immunodeficiency virus 1 infections and, more recently, SARS-CoV-2 [19,20]. In this latter case, SARS-CoV-2 binds to platelets probably via its receptor ACE2, and viral hemagglutinin is cleaved by TMPRSS2 to activate the internalization of the virus. Such cleavage triggers platelet activation and downstream signaling events leading to cytokine overproduction, platelet aggregation, and leukocyte–platelet aggregate formation and, ultimately, to the more severe spectrum of COVID-19 characterized by cellular damage, acute lung injury, and thromboembolism [21].

During infections, platelets are a crucial mediator of the inflammatory response activating and initiating their antimicrobial host defense by sensing the presence of pathogens or inflammation through immune receptors such as immunoglobulin or complement receptors and TLRs [22]. Interestingly, platelets recirculate after degranulation and sequestration, demonstrating that in adaptive immunity their role is proactive and longer-lasting [23].

## 3. Conclusions

Clinical observations from several infectious diseases including COVID-19 and Long Covid, and recent basic science discoveries clearly suggest that ECs and platelets play a pivotal role in immune responses, chronic inflammation, and, possibly, long-term outcomes in infections and other inflammatory diseases. The most recent data derive from COVID-19 and Long COVID and showed that anticoagulation and/or anti-aggregation approaches improved both outcomes in severe COVID-19 and improved symptoms in Long COVID patients, further highlighting fascinating perspectives on targeting the ECs and platelets in patients with infectious conditions and suggesting that controlling inflammation alone may not be sufficient to completely restore homeostasis in chronic inflammatory processes.

Importantly, the role of ECs and platelets in modulating immune responses has been less studied in children. This is a gap that needs to be filled soon given growing evidence from adult studies for their disease-contributing role. The pathophysiology of platelet and EC involvement in COVID-19 may be different between children and adults. Future research should not be limited to exploring the role of platelets and ECs on blood clotting and vascular homeostasis, but also in their more complex involvement in immunity and as targets for future anti-inflammatory therapies. 

## Data Availability

Not applicable.
